# The NIST Length Scale Interferometer

**DOI:** 10.6028/jres.104.017

**Published:** 1999-06-01

**Authors:** John S. Beers, William B. Penzes

**Affiliations:** National Institute of Standards and Technology, Gaithersburg, MD 20899-0001

**Keywords:** graduated scales, interferometry, length, measurement assurance, measurement uncertainty

## Abstract

The National Institute of Standards and Technology (NIST) interferometer for measuring graduated length scales has been in use since 1965. It was developed in response to the redefinition of the meter in 1960 from the prototype platinum-iridium bar to the wavelength of light. The history of the interferometer is recalled, and its design and operation described. A continuous program of modernization by making physical modifications, measurement procedure changes and computational revisions is described, and the effects of these changes are evaluated. Results of a long-term measurement assurance program, the primary control on the measurement process, are presented, and improvements in measurement uncertainty are documented.

## 1. Introduction

### 1.1 Importance of Length Measurement

Major changes have occurred in dimensional metrology in response to rapidly increasing scientific and industrial accuracy requirements over the past several decades. Accurate measurements of all kinds are vitally important to many scientific and industrial operations, and measurement of length is fundamental. Measurement of physical objects for quality control and process control is part of this picture. Semiconductor manufacturers, for example, have achieved remarkable improvements in quality control on ever smaller circuitry as a result of improved metrology in mask fabrication and production processes. Linear rules in many forms from encoded scales to photo lithographic grids are often used as check standards for such purposes and must be calibrated, when usage requires, to relate them to the international standard of length. For more than thirty years the evolutionary development and performance of the NIST length scale interferometer has influenced improvement in high accuracy length measurements.

### 1.2 Changes in the Meter Definition

In 1960 the Conférence Générale des Poids et Mesures (CGPM) adopted a new definition of the meter specifying it as an exact number of vacuum wavelengths of a particular spectral line of the ^86^Kr isotope. This replaced the prototype platinum-iridium meter bar kept at the Bureau International des Poids et Mesures (BIPM) in Sévres, France as the international definition since 1889. Again in 1983, the CGPM redefined the meter, this time as the path length traveled by light in vacuum in 1/299792458 second [1]. In both redefinitions great pains were taken to make as little change as possible in the actual length of the meter. Along with the 1983 action, the CGPM sanctioned several vacuum wavelengths of iodine stabilized helium neon lasers as working length standards for relatively short lengths. Time-of-flight measurements are generally impracticable for lengths less than 50 m.

### 1.3 Changes in Length Measurement Methods

From 1889 to 1960, the international prototype length standard and the national length standards were graduated platinum-iridium meter bars. Intercomparisons of national standards with the prototype bar and calibrations of all other scales were accomplished in comparators employing filar microscopes for measuring length differences between a standard and an unknown [2]. Subinterval lengths were determined in the same comparators by a lengthy process of intercomparison by subdivision [3].

In 1958, NIST, then the National Bureau of Standards (NBS), began designing an interferometric line scale comparator in preparation for the proposed change in the definition of the meter. The design was based on the development of high speed automatic interference fringe counters in the preceding decade, opening a new era of length measurement by interferometry.

Prior to 1960, wavelengths from selected atomic light sources were sanctioned by CGPM as secondary realizations of the meter. These sources were used in measuring end standards of length. End standards are bars, usually metal, with optically finished, plane, parallel end faces defining a length. Measured by static interferometry^1^ since early in the 20th century, their gaging faces are ideally suited to this method because they can serve as mirrors in the measuring legs of interferometers. Line scales cannot be measured efficiently by the static method for a number of reasons including the multiplicity of subintervals. They are best measured by dynamic (displacement) interferometry.

### 1.4 Varieties of Scales

Graduated length scales come in many forms, and are made in lengths from a few micrometers to over a meter. Those longer than a meter or two are usually classified as measuring tapes or rods. Many materials are used including steel, Invar, glass, glass-ceramics, silicon, and fused silica. Cross sectional shape can be rectangular, “H,” modified “U” (flat bottom), or a modified “X” (Tresca). At present, the line scale interferometer is limited to graduation widths ranging from sub-micrometer to 100 μm, and spacings ranging from less than 1 μm up to 1025 mm. Spacings are generally measured from center to center of the graduations, but can also be measured from edge to edge.

Some devices that are not strictly linear scales are measured in the line scale interferometer. These include end standards in a size range (250 mm to 1000 mm) that can present measurement problems with static interferometry. They are converted into line scales with ruled auxiliary blocks wrung to each end [4]. Once converted, the distance between the graduations is measured and then the auxiliary block line spacing is subtracted to arrive at the length of the end standard. Grid plates are measured by treating each row and column of graduations as an independent scale and, when possible, an estimate of orthogonality can be made by measuring the diagonals.

## 2. The NIST Length Scale Interferometer

### 2.1 Design and Evolution

**Fig. 1:**
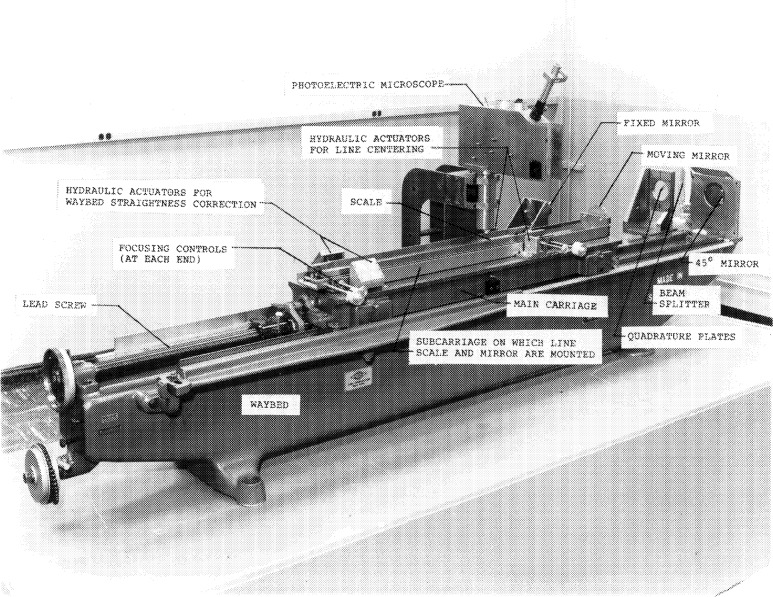
The length scale interferometer mechanical and optical components prior to 1970.

By 1961 an experimental Michelson type fringe counting interferometer using a ^198^Hg light source as a length standard was tested [5], but restricted coherence in atomic sources limited the range to about a decimeter. Development of a final version capable of measuring length scales proceeded, by good fortune, in parallel with the development of lasers [6]. The remarkable coherence of laser light made it ideal for fringe counting interferometry over long distances. In 1965, the two activities converged and a successful measurement of a 1 m scale with helium-neon laser wavelengths was accomplished [7]. Further refinements were then made in the line scale interferometer and laser wavelength stability was improved. By 1966, a semi-automated length scale measurement system was in operation [8]. The mechanical and optical structures as they existed at that time are shown in Fig. 1.

Development has continued on both the line scale interferometer [9] and on lasers [10, 11]. The major changes were: (1) rigid coupling and kinematic mounting of the microscope and interferometer fixed leg in 1970, (2) replacement of the Michelson-type plane mirror interferometer with a commercial laser interfer ometer in 1979, (3) modernization of the microscope electronics together with the addition of a computer/controller in 1981 to completely modernize and automate the instrument [12].

Many minor modifications in the mechanical structure were also made as well as important changes in measurement procedures, data processing, and computational methods. These will be discussed under measurement assurance (Sec. 3).

### 2.2 Description

The major components of the instrument are:

1. A 2 m waybed with a lead-screw driven carriage for translating the scale.
2. A fixed position photoelectric microscope with a servo system for centering scale graduations in the microscope field.
3. An interferometer using a stabilized laser light source as a length standard for measuring scales.
4. Measuring devices for temperature, barometric pressure, and air moisture content for use in computing laser wavelengths at ambient conditions.
5. A computer/controller for data recording and processing, and for automated instrument operation.
6. A temperature controlled enclosure to provide a stable environment for the instrument.

#### 2.2.1 Mechanical Structure

The 2 m waybed and its main carriage, originally a linear dividing engine made by the Société Genevoise d’Instruments de Physique (SIP)^2^, was modified at NBS for use as the mechanical foundation of the length scale interferometer. The main carriage has precision roller bearing wheels and rides on hand scraped guide ways. It is moved by a 1 mm pitch, 1025 mm long, lead screw.

Since modification, the main carriage is coupled to the lead screw by a nylon half-nut and the screw is driven by a computer controlled stepping motor for coarse scale positioning. The weighted half-nut rides on top of the screw to permit easy decoupling for manually and quickly moving the carriage back and forth during scale and interferometer alignment procedures.

As shown in Figs. 2a and 2b, a superstructure is mounted atop the main carriage to provide a scale mounting platform. Mechanical controls for focusing and aligning the scale, and a servo actuator for centering scale graduations in the microscope field, are also provided. Line centering is accomplished by a servo signal from the photoelectric microscope to a pair of hydraulic pressure actuators, one on each side of the superstructure. Pressure is applied to flexure plates that support the superstructure at the right end. A pair of cables suspend the structure at the left end so it is free to move several micrometers along its axis as pressure is applied in the line centering process.

The scale mounting platform is a 110 cm × 5 cm × 0.95 cm Invar plate with a retroreflector attached to each end, serving as the moving elements in two separately or simultaneously operating interferometer systems. Each system has its own laser and counter. A fixed position remote interferometer, consisting of a beam splitter and a cube corner retroreflector in a compact assembly, is located near each end of the waybed. Each remote interferometer splits its laser beam into two beams of equal intensity. One beam forms a reference leg within the contiguous retroreflector, and the other forms a measuring leg with the moveable retroreflector.

One remote interferometer and the microscope are attached to opposite ends of a 1075 mm rigid steel tube and this assembly is mounted over the waybed on a three point kinematic support. The right end of the assembly (with its remote interferometer) is fixed to the waybed. The left end (with the microscope) is near the waybed midpoint. It rests on a ball between two flats on the far side of the waybed and a ball between a flat and a “V” groove parallel to the ways on the near side. A second remote interferometer is at the extreme left on an extension of the steel tube. If the microscope and interferometers were rigidly and independently attached to the waybed, they would be shifted by the heavy carriage as it moves on the waybed. Rigid interconnection and kinematic mounting of these components frees them from this shifting.

It is vital that the distance between the microscope and the remote interferometers remains fixed during a scale measurement because any relative motion between them will be seen as part of the measured scale length, thus obviously resulting in uncertainties. By the same token, it is vital that the dimension between the scale and the retroreflectors remain fixed during a measurement.

The entire apparatus is housed in a vibration isolated and thermally insulated enclosure held at 20 °C by a thermostatically controlled circulating water system. A plywood box forms the outside of the enclosure. It is lined with plastic foam insulation and a sheet copper inner lining having copper tubing soldered to it for carrying the thermostated water. The upper half of the enclosure consists of three raisable sections providing access to the instrument.

#### 2.2.2 Laser Interferometer

**Fig. 3:**
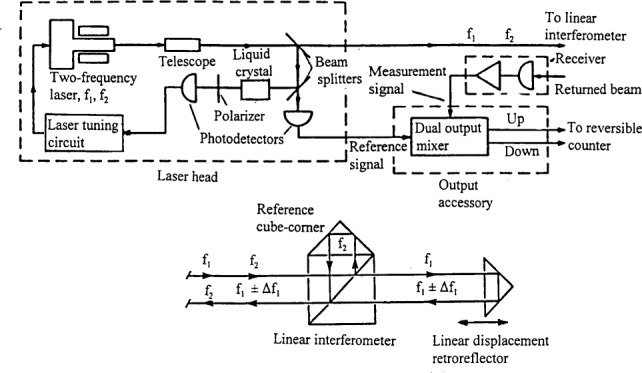
The Hewlett-Packard interferometer system.

A Michelson-type, plane mirror interferometer was used in the instrument until 1979. It was replaced with a Hewlett-Packard (HP) model 5526A He-Ne laser interferometer. Elements of the HP interferometer are shown in Fig. 3. It can be readily integrated into an automated, computer controlled system. Aligning the optical components is much simpler with the HP system. However, the ability was lost to compensate for waviness and deviations in straightness of the ways provided in the Michelson by a servo system [12] that kept the optical flats parallel to each other as the carriage moved. To minimize sine and cosine measurement uncertainties introduced by carriage pitch (from waviness) and yaw (from straightness deviations), the optical axis of the HP interferometer is always aligned in coincidence with the scale graduation axis.

The Michelson and the HP system were operated simultaneously from opposite ends of the waybed during a transition period to verify comparability. An iodine stabilized He-Ne laser was used with the Michelson during periods in 1976 and 1978 to confirm wavelength values of the Lamb-dip stabilized He-Ne laser in use at the time. Short term fractional stability of the HP laser wavelength was 1 × 10^−8^, and long term fractional stability was 1 × 10^−7^. Long term fractional stability of the iodine stabilized laser was 1 × 10^−10^.

The HP interferometer is operated in a 1/4 wavelength counting mode with interpolation between counts. Measured values of air temperature, barometric pressure, relative humidity, and CO_2_ content are used to calculate the refractive index of air in the interferometer path. Ambient wavelength is then derived from the NISTcalibrated vacuum wavelength and the refractive index to make scale length computations.

#### 2.2.3 Photoelectric Microscope

Line scales are calibrated by making carriage displacement, as measured by the interferometer, correspond exactly to scale interval lengths. The key to this correspondence is the line centering action of the photoelectric microscope that occurs when the carriage is stopped and a graduation is in view. This process is described below.

The high speed stepping motor and its indexer are interfaced with the system computer/controller to move the carriage predetermined distances corresponding to nominal line spacings on the scale. During final indexing steps for each interval the stepping motor goes into a slow mode and actuates microscope electronics that trigger a stop-motor signal when the line appears in the microscope field. Once the carriage is stopped, line centering electronic circuits take over. The graduation is then centered in the field by means of electro-hydraulic actuators on the scale support structure. Therefore, starting at the zero graduation, and ending at the terminal graduation, the interferometer measures the distances from line center to line center of pre-selected intervals, thus calibrating the scale. In actual use, several passes are made up and down the scale to obtain redundancy needed for averaging and statistical analysis.

**Fig. 4:**
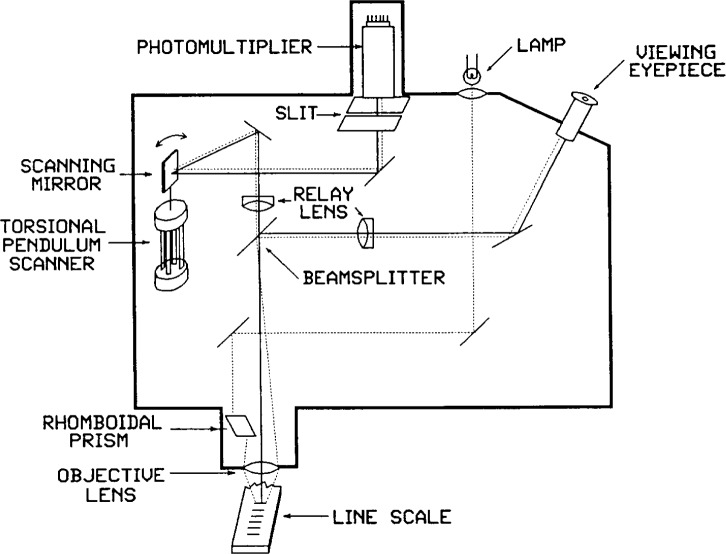
Scanning photoelectric microscope schematic diagram.

In both design and operation the photoelectric microscope is the most complex part of the length scale interferometer. A schematic diagram of the microscope optics is shown in Fig. 4. An oscillating mirror sweeps a magnified image of the vertically illuminated scale graduation segment across a slit in front of a photo multiplier. A sinusoidal scanning mode is used rather than a linear mode to avoid all the harmonics of the scanning frequency. The torsional-pendulum scanner has a very high vibrational Q of 1000 and has a resonant frequency of 70 Hz. Since the scan has only one frequency, and the scanner is mechanically resonant, its center of oscillation is very reliable and has a close and fixed relation to the microscope optical center. This is extremely important to microscope operation because the line center is locked to the oscillation center when interferometer readings are made, and the oscillation center is the operational center of the microscope field.

Scale graduations are of two types as seen by the microscope: bright (reflective) lines against a dark background, or dark lines against a bright background. Graduation images are produced with the aid of a microscope illuminator which projects a uniformly lit beam of light through the objective lens and normal to the scale surface. If graduations are chrome on a transparent substrate the line will reflect light and appear bright on a dark background. The reverse of this will appear if the background is chrome and the line is transparent. On a metal scale the surface is flat and highly polished, and the lines are cut into the metal with a diamond ruling point producing a dark line on a bright background. The line appears black because the illuminating light is scattered by the sloped sides of the V-shaped groove cut by the ruling point. Some ruled lines are partially filled with printers ink to enhance their darkness and avoid reflections from the flat bottom of the groove. High quality lines are uniform in width and have smooth sharp edges. Typically, they come in widths from 1 μm to 10 μm.

Several electronic circuits are employed in operating the microscope. These are a mirror drive oscillator, optical signal processor, line center deviation detector, indexer control interface, and a proportional plus integral servo driver. The line signal derived from the photo multiplier is displayed on an oscilloscope operating in the *x–y* plot mode. The horizontal input of the scope, representing the scan, is driven by the sinusoidal signal from the mirror drive oscillator circuit, while the vertical axis represents light level. Thus, a linear signal is displayed on the screen showing the line profile and its position relative to the scan (microscope) center.

**Fig. 5:**
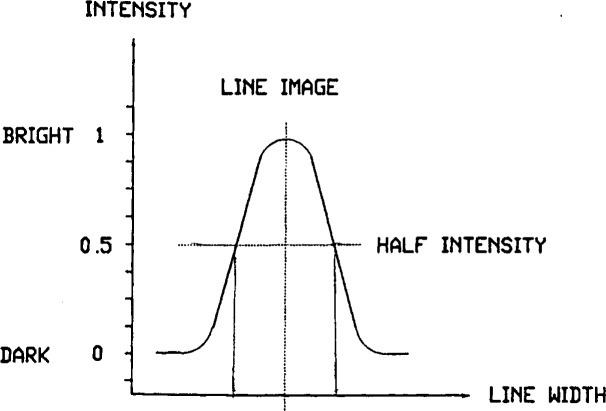
Microscope line signal pattern.

Optical signal processor and line center deviation detector circuits transform the signal into a means for line centering. A pair of positive and negative peak detectors make up the optical signal processor. The difference between positive and negative peak voltages represents the deviation of the line center from the scan center. A threshold voltage derived from the scaled amplitude of the line signal is displayed on the oscilloscope as a horizontal line intersecting the line signal pattern (see Fig. 5) and is used in the line signal deviation detector to produce a dc signal proportional to the distance of the line center from the scan center. The sign of this signal shows the direction of the line from center. Threshold voltage is adjusted with a potentiometer to an optimum level, usually 50 % of the amplitude, for triggering the centering-deviation detector. Using the two points where the threshold voltage intersects the line signal, the line centering deviation detector generates a bipolar dc voltage corresponding to the direction, and proportional to the distance, of the line from center. This voltage is used as a correction signal to run the proportional plus integral servo driver which then moves the scale support structure (with retroreflector attached) by electro-hydraulic means to center the line. This servo system loop will continuously and dynamically lock a line center onto the scan center of the microscope field. Centering of a line edge can be achieved using the intersection of the threshold with one edge or the other of the line signal, a useful feature for special measurements where edge to edge rather than center to center measurements are required.

From the preceding description it is clear that the microscope is an edge detector and when it sees both edges of a line at the same time it will find and use its center. Although this “two point criterion” for determining line centers was chosen for operational reasons, it is remarkably similar in function and result to the bi-filar eyepieces used in microscopes employed in traditional line scale metrology. The bi-filar microscope has a pair of parallel hairs in the eyepiece assembly slightly farther apart than the width of the graduation image. These hairs are moveable in unison and their position across the field of view is readable by a micrometer screw with a graduated drum and a drum revolution counter. When viewing a scale, the line center is located by straddling the line with the double hairs and moving them until the open areas on each side of the line between the line edges and the hairs are equal. This process takes advantage of a human vision characteristic called hyperacuity which permits the balancing of areas much more precisely than would seem theoretically possible from the often erroneously applied criterion of lens resolving power. With the hyperacuity phenomenon line centers can be located in a bi-filar microscope, with a standard deviation of 40 nm. However, the photoelectric microscope can locate line centers to better than a 2 nm standard deviation.

#### 2.2.4 Temperature Control and Measurement

By international agreement precision length measurements are made near, and corrected to, a temperature of 20 °C.

The line scale laboratory is in a below-grade room to help isolate it from vibration and outdoor temperature fluctuations. Laboratory temperature is controlled at 20 °C ± 0.05 °C by a thermostated forced air heating/cooling system. Tempered air is distributed evenly through a plenum formed by a dropped and perforated false ceiling. Air exits at baseboard level and returns through hollow walls and ducts to the tempering system.

**Fig. 6:**
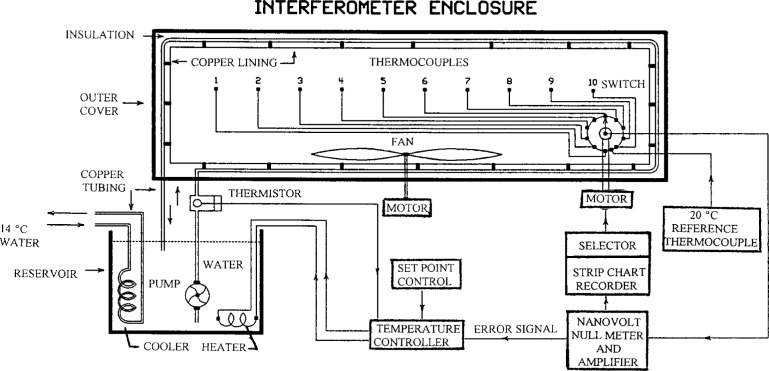
Temperature control system schematic diagram.

The interferometer enclosure is independently and precisely temperature controlled to provide the environment needed for accurate length measurements. Figure 6 is a schematic diagram of the temperature control and measuring system for the interferometer enclosure. Distilled water in a reservoir is cooled at a constant rate by a heat exchanger carrying chilled water at 14 °C. A submerged electric heater proportionally heats the distilled water to keep it at the 20 °C set point. A pump circulates the water through copper tubing behind the copper lining of the housing.

When the temperature system is activated for making a length measurement an error signal comes from a thermocouple in the interferometer air path to give precise control at 20 °C ± 0.005 °C, but when the temperature system is not activated the error signal comes from a thermistor in the water line to keep the temperature nominally correct when the instrument is not in use.

An 0.5 m diameter fan is located on the enclosure bottom to circulate air and reduce temperature gradients. All heat producing elements such as the laser, microscope illuminating lamp, lead screw drive motor, fan motor, and electronic circuits are externally mounted.

**Fig. 7:**
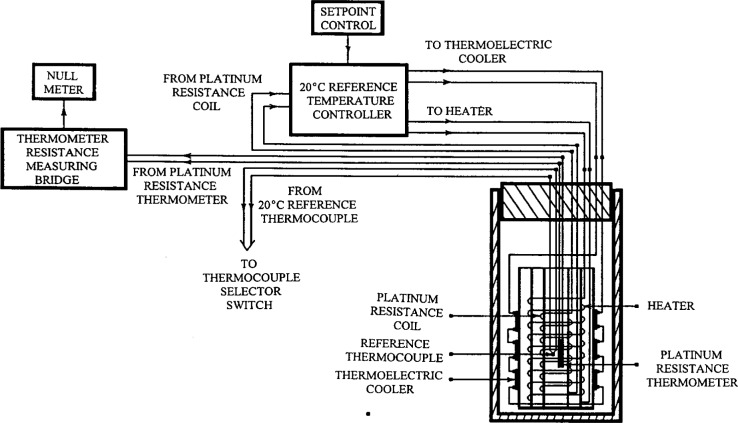
Reference temperature measuring and control system schematic diagram.

At the heart of the temperature control and measurement system is a 20 °C reference temperature cell and a calibrated standard platinum resistance thermometer (SPRT). A schematic of the system is shown in Fig. 7. The cell provides a precise 20 °C ± 0.0005 °C reference for the thermocouples in the temperature measuring system. Cell operation is based on the same principles as the other control systems. It has a constant source of cooling, provided in this case by a thermoelectric cooler, modulated by feedback to an electric heater to keep a constant temperature.

Cell temperature is monitored with the SPRT by keeping the Mueller-type bridge set at the 20.000 °C resistance value. There is some drift in the reference cell heater current controller, but a manual adjustment to the control circuit potentiometer once a day keeps the cell temperature stable to better than 10^−3^ K.

There are 10 copper-constant thermocouple junctions inside the enclosure, with their reference junction attached to the SPRT in the reference cell. Two are in the interferometer air path, three are on the scale being measured, and the rest are in various locations where they are used to detect gradients and temperature variations. A motor-driven selector switch allows temperatures to be read sequentially and displayed in degrees Celsius on a strip chart recorder. There are two operating modes for the system: control and measurement. In the control mode the selector switch is locked on one thermocouple in the interferometer air path. Its voltage indicates the deviation from 20 °C and is the error signal that feeds back to the water bath temperature control to correct the air temperature. The measurement mode is used periodically during a scale measurement by unlocking the selector switch to read air path and scale temperatures for use in computing scale length.

This system has several advantages. Thermocouples are very stable and are free from self heating. Thermocouple voltage measurement uncertainties are minimized because the measuring junctions are at nearly the same temperature as the reference junction making thermocouple voltages very small and therefore measurable to high accuracy. In addition, the SPRT and the Mueller bridge are quite stable and do not require frequent calibration. Finally, the system is directly traceable through the SPRT to the International Temperature Scale of 1990 (ITS-90).

#### 2.2.5 Atmospheric Pressure Measurement

Barometric readings are taken with electro-mechanical pressure transducers having a sealed chamber with a low stress diaphragm on one wall. As the diaphragm moves with changes in atmospheric pressure, its position is detected with a capacitance probe that produces a voltage proportional to pressure. Two barometers of different manufacture are constantly checked against each other to detect drift that may indicate a need for recalibration. NIST calibrations establish the exact relationship between pressure and voltage.

#### 2.2.6 Water Vapor Control and Measurement

To reduce the possibility of rust forming on steel surfaces, laboratory relative humidity is held below 50 % by the air conditioning system. Water vapor content of the air in the interferometer housing is measured with a NIST calibrated, chilled mirror type, dew point hygrometer. A second similar hygrometer serves as a check on the primary instrument. An instrument based on capacitance change in a thin polymer film as it absorbs or evaporates water from the air provides a third reliability check.

#### 2.2.7 Computer/Controller

A Hewlett-Packard desktop computer controls the operation of the line scale interferometer system. It takes operator instructions, controls the carriage-driving stepping motor, records line scale measurement data, computes scale interval lengths, and prints a calibration report table complete with statistical analysis and measurement uncertainties.

**Fig. 8:**
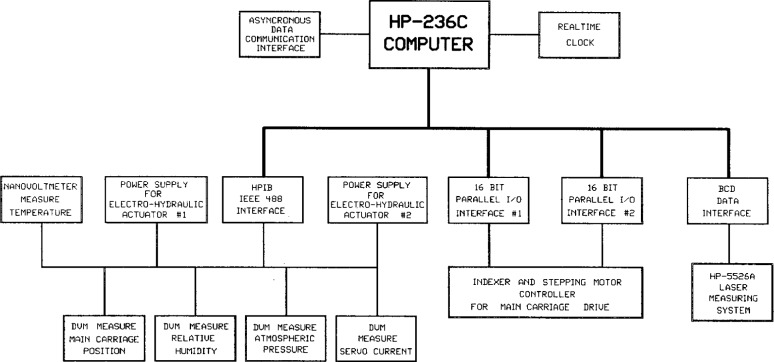
Computer interfaces for the length scale measuring system.

Figure 8 is a schematic diagram of the computer and its interfaces. There are two 16 bit I/O interfaces: one controls the stepping motor and the other controls the line centering process. The laser interferometer display unit is connected to the BCD interface to receive quarter wavelength counts and the interpolated fractional count.

Several instruments are connected to the HP-IB interface. A digital voltmeter (DVM) measures the line centering servo current, a second DVM measures the output from a hygrometer, and a third measures the output from a barometer. A nanovoltmeter measures the thermocouple outputs to give air and scale temperatures.

### 2.3 Operation

A scale to be measured is first mounted on the carriage with its zero graduation at the left (called normal scale orientation). As the carriage is manually moved back and forth the scale is aligned and focussed relative to the microscope, then the interferometer is aligned coincident with the scale graduation axis. At the end of this procedure the carriage is positioned so the zero scale graduation is in the microscope field. After the temperature stabilizes at or very near 20 °C, and temperature gradients are minimized (usually overnight), the scale is ready for measurement. A computer program governs the measurement process. Preliminary data that must be entered by the computer operator includes scale identification, scale thermal expansion coefficient, data file name, the intervals to be measured, and the number of measurement passes to be made. Interferometer path air temperature and relative humidity, scale temperature, and barometric pressure can be entered by the operator or read automatically. Although set to a nominal zero at this point, the interferometer always displays a fractional count which is read by the computer at the start of the measurement.

The computer starts the measuring process as soon as preliminary data are entered. It verifies that a graduation is present and centered in the photoelectric microscope field, and reads the initial interferometer count. It also calculates the steps required of the stepping motor to reach a point just short of the next programmed graduation, and starts the stepping motor in its high speed mode. When the required steps are reached the motor shifts into its low speed mode and the microscope electronic circuits are activated. As the graduation enters the microscope field the line detection circuit stops the stepping motor and activates the hydraulic line centering servo system. When the line is centered, the interferometer is read. This process is repeated for each succeeding equally spaced graduation until the terminal graduation is reached. The operator enters a reduced list of preliminary data for the return pass and the process reverses until it closes at the zero graduation. More passes are performed as needed.

The scale is then remounted on the carriage with the zero graduation at the right end (reversed orientation), and the process is repeated as for the normal orientation. Two passes in each orientation is the minimum to provide valid statistical information. Increasing the number of passes generally reduces standard deviation and, consequently, uncertainty.

### 2.4 Length Computations

Data from the measuring system is converted into scale interval lengths at 20 °C by the computer program. The count recorded at the zero graduation is subtracted from each succeeding recorded count in the pass to give the interval lengths in quarter wavelength units. A fringe multiplier then converts these into scale length units at 20 °C.

#### 2.4.1 Fringe Multiplier

Three elements are combined in the fringe multiplier: (1) laser vacuum wavelength, λ0, (2) refractive index of ambient air in the interferometer, *n_tpf_*, and (3) scale expansion, Δ*L* from observed temperature to 20 °C. Laser vacuum wavelength is determined by NIST calibration.

Until 1991 the refractive index of air was computed from Edlén 1966 equation [13]. Birch and Downs [14] recently developed a modified form of the equation that provides significant improvement in uncertainty [15]. The changes are mainly in the correction for air moisture and in values for CO_2_ content of air. A mass fraction value of 300 × 10^−6^ of CO_2_ is used in the original Edlén equation. Birch and Downs use 450 × 10^−6^ as typical of their laboratory air, but in the formula below the measured value of 380 × 10^−6^ for the NIST line scale laboratory is used [16]. The modified formula is shown below. Symbols are defined and units are specified for all equations in this section in the *Key to symbols and units* table at the end of the section.

$$n_{tpf}=1+AB-C$$ (1)

where

$$A=\frac{p\left[8342.22+240\;6057{\left(130-\sigma^{2}\right)}^{-1}+15997{\left(38.9-\sigma^{2}\right)}^{-1}\right]10^{-8}}{96095.43}$$ (2)

$$B=\frac{1+p\left[0.601-0.00972t_{a}\right]10^{-8}}{1+0.003661t_{a}}$$ (3)

$$C=f\left[3.7345-0.04.1\sigma^{2}\right]10^{-10}$$ (4)

Wavelength in ambient air is then determined from the fundamental relationship

$$\lambda_{tpf}=\frac{\lambda_{0}}{n_{tpf}}.$$ (5)

Scale expansion at temperature *t*_s_ relative to 20 °C is expressed by

$$\Delta l=\alpha l\left(20{}^{\circ}C-t_{s}\right).$$ (6)

Wavelength in ambient air and scale expansion are combined in the fringe multiplier *M*. For the HP interferometer, which counts in quarter wavelengths, we have

$$M=0.25\lambda_{tpf}\left[1+\alpha \left(20\;{}^{\circ}C-t_{s}\right)\right]10^{-3}$$ (7)

and

L=L′M, (8)

where *L* is the length of the measured scale interval in mm at 20 °C, and *L'* is the number of quarter wavelengths (λ*tpf*/4) corresponding to the scale length.

##### Key to Symbols and Units

*t*_a_: Temperature in the interferometer air path in °C
*t*_s_: Temperature of the scale in °C.
*p*: Atmospheric pressure in Pa.
*f*: Water vapor pressure in Pa.
*n_tpf_*: Refractive index of air at observed *t*_a_, *p*, and *f*.
*α*: Linear thermal coefficient of expansion ofthe scale.
*λ*_0_: Vacuum wavelength of laser in mm.
*λ_tpf_*: Laser wavelength at observed *t*_a_, *p*, and *f*.
*σ*: 1/*λ*_0_ in μm^−1^.
*1*: Nominal scale interval length in mm.
*L′*: Measured length of scale interval in interference counts (quarter wavelengths).
*L*: Measured length of scale interval in mm at 20 °C.

#### 2.4.2 Measurement Data Presentation

A calibration report is produced by the computer for measured scales. Included in the report are an explanatory text, a table of results, and a graph as shown in Figs. 9a and 9b.

## 3. Measurement Assurance

### 3.1 Definition and Purpose

Measurement assurance is a systematic program employing redundant measurements of stable control standards for continually monitoring a measurement process and determining its performance parameters.

Treating regularly performed measurements as a production process is the basis of the program, while statistical analysis and control charts provide means for characterizing the process. Any organized measurement procedure that generates statistically significant amounts of data can be treated as a production process, monitored with controls, and analyzed to reveal characteristics such as measurement variability and uncertainty.

This approach to measurements was pioneered by Churchill Eisenhart [17] in the 1960s. Its applications were developed and expanded by Paul Pontius [18], Joseph Cameron [19] and Carol Croarkin [20] among others.

In a complex measurement system, such as the line scale interferometer, it is essential to know that all components are working properly. Substantial uncertainties can enter the process if, for example, the interferometer is misaligned, the barometer is out of calibration, or the distance between the remote interferometer and microscope changes during a measurement. A measurement assurance program (MAP) with its control standards, control charts, and statistical analysis is indispensable for detecting process uncertainties and malfunctions. It is also very useful for measuring effects of planned changes made for process improvement.

### 3.2 Control Standards

Two primary control standards are used to monitor this process. The first control, introduced in 1966, is a SIP Invar meter bar, M5727. It has an H shaped cross section with its neutral plane, located on the upper surface of the H cross piece, graduated in millimeters. The second control, introduced in 1982, is an 0.508 m (20 in) steel scale, No. 6495, graduated each 1.27 mm (0.05 in). A measurement process such as this one, which runs for decades, should have dimensionally stable control standards but completely stable materials are very rare, perhaps even non-existent. This can make it difficult to distinguish real from apparent secular length changes in the controls.

### 3.3 Process Changes and Control Charts

**Fig. 10:**
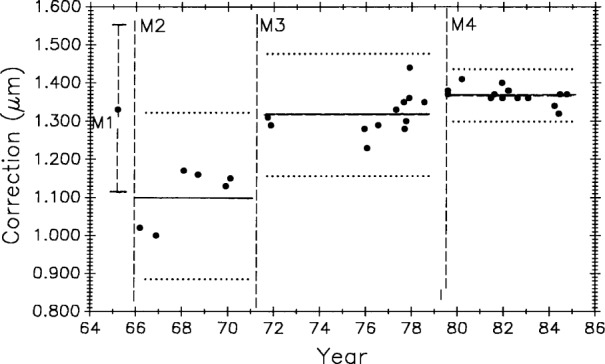
Meter M5727 control chart, 0 m to 1 m, 1971 to 1991.

Figure 10 is the control chart showing the measurement history of M5727 (0 m to 1 m interval) from 1965 to 1985. Individual measurement values are plotted. A change in the measurement process was made at each vertical line, dividing the plot into periods. The mean value for each period is shown as a horizontal solid line, and the limits shown with dotted lines are ± 3*σ* where *σ* is the standard deviation of a single value.^3^ The ± 3*σ* control limits are used to judge individual measurements. They are a statistical prediction, based on past performance, that 99.7 % of future measured values will fall within them. Values falling outside these control limits are judged to result from an out-of-control condition and require an investigation into the cause and a correction of the fault. The 3*σ* level is a generally accepted value for judging performance in measurement assurance programs.

Each process change and the mean value for its period is designated by M1 through M4. The change associated with each mean is described in Table 1. Data taken and changes made after 1985, designated as M5 through M8, are also described in the table but will be dealt with later in the text.

An individual measurement is defined here as the mean of two data sets of two passes each. One set is taken with the bar in the normal orientation (zero graduation at the left) and consists of one pass up the scale from zero to the terminal graduation (one meter in this case), and one pass down the scale, closing at the zero graduation. Data stops are made at each decimeter in both directions. The second set consists of two passes taken in a similar manner but with the bar in the reversed orientation (zero graduation at the right).

Two critical interferometer dimensions are given special attention in making process changes. These are: (1) distance between microscope and interferometer beam splitter, and (2) distance between retroreflector and scale. Changes in these relationships during a scale measurement will cause uncertainties.

### 3.4 M5727 Control Data, 1965 to 1985

Table 2 shows the mean value and long term measurement variability, as represented by control chart limits, for each period shown graphically in Fig. 10.

The M1 period measurements were made in 1964–65 by comparing M5727 with NBS laboratory reference meter bars in a comparator employing micrometer microscopes to measure the length differences between reference bar and unknown in the traditional way [2, 3]. The mean value is plotted in Fig. 10 with its 3*σ* control limits. The first interferometric measurement period, M2, extended from 1966 to 1971. The M2 mean agreed with M1 within 0.2 μm. This was considered to be good agreement for that time when uncertainties for lengths up to 1 m were in the range of 0.2 μm to 0.5 μm.

A major structural change in the interferometer was completed in 1971. The photoelectric microscope was originally attached to the side of the waybed at the midpoint, and the beamsplitter was attached to the waybed at one end (see Fig. 1). A steel I-bar (not shown in Fig. 1) extending from the microscope to the top of the beamsplitter assembly was installed very early to connect more rigidly these two components when it became apparent that the microscope angle changed when the carriage moved on the ways. The beam reduced this distortion, but a more effective structure was designed and installed in 1970–71.

In this new structure the microscope and beamsplitter are mounted at opposite ends of a 1 m steel tube and the assembly kinematically mounted on the waybed. The beamsplitter end is fixed to the end of the waybed and the microscope end is supported on two 25.4 mm ball bearings free to roll only in a direction parallel to the ways (see Fig. 2). This decoupled the assembly from waybed distortions. Period M3 shows resulting measurements. There was an improvement in variability and a change in apparent length.

By 1978 the original fringe counting and microscope electronics were failing from age. In 1979 the electronics were replaced with modern components and the original NBS-made, Michelson-type, interferometer was replaced with a commercial model. All this was done while preserving the principles of the original NBS design. Commercial laser interferometers were by then quite reliable so a Hewlett-Packard (HP) model was installed. HP interferometer alignment was easier and more precise than with the Michelson, and the resulting improved measurement variability can be seen in period M4.

In the 1986 evaluation [20] of M1 through M4 data, a possibility of real secular lengthening of M5727, as suggested by the slope of a fitted line, was considered. It was rejected for several reasons. Most important was that the first interferometric measurement period, M2, was the least reliable, having been taken before the fixed optical components of the interferometer and the microscope were rigidly coupled and kinematically mounted. The change in mean value from M2 to M3 was, therefore, discounted as evidence for dimensional change in M5727. The relatively small change in M5727 from M3 to M4 was not statistically significant, but it might be attributable to measurement process change M4 (replacement of the Michelson type with an HP interferometer). However, the two interferometers were operated simultaneously, from opposite ends of the waybed, for a short period in 1979 to verify continuity. They agreed to within 0.01 mm in the measurement of M5727.

### 3.5 Control Data, 1971 to 1991, A New Process

#### 3.5.1 Meter Bar M5727

**Fig. 11:**
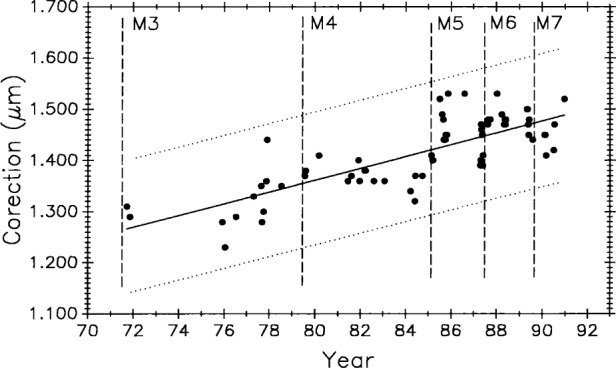
Meter M5727 control chart, 0 m to 1 m, 1971 to 1991.

Figure 11 shows M5727 measurement history to 1991 with M1 and M2 data deleted to eliminate influence from biased early data. Starting with period M3 the data are treated as a new process reflecting major structural and operational changes that produced improvements in measurement results. Vertical lines correspond to process changes M5 through M7 in Table 1 (M8 data will be shown and discussed later). Horizontal lines are period mean values. This additional data strengthens the case for a gradual lengthening of this bar.

#### 3.5.2 Scale No. 6495, 508 mm (20 inches)

**Fig. 12:**
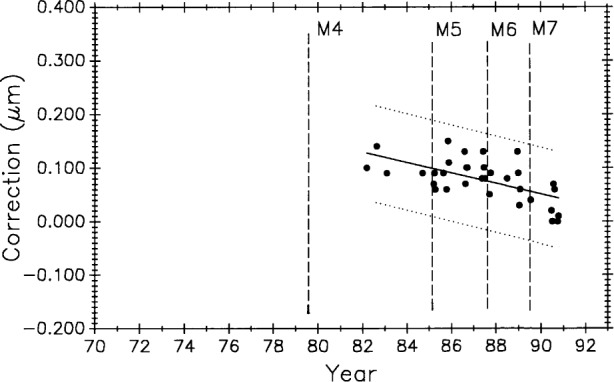
Control chart for bar No. 6495, 0 m to 0.508 m, 1982 to 1991, plotted on same time scale as Fig. 11.

Figure 12 is the history of steel control bar No. 6495 plotted on the same time scale as meter bar M5727 in Fig. 11. During the time common to both scales, the length of the former appears to be increasing and the latter appears to be decreasing.

### 3.6 Evaluation of Measurement Uncertainties

The 1986 uncertainty evaluation (see column labeled Process relative standard uncertainty, 1986 in Table 3) was based on data up to 1985. In 1987, a re-evaluation of measurement process uncertainties was undertaken. Although control standards are indispensable tools for monitoring the measurement process, they have the disadvantage of undergoing long-term length changes, leaving some doubt about process performance. Consequently, three goals were set for the new uncertainty study: (1) reduce measurement uncertainties, (2) establish a new measurement uncertainty value by approved methods [21], and (3) determine rates and directions of control bar length changes.

#### 3.6.1 Uncertainty Sources

The following is a list of potential length scale measurement process uncertainty sources:

- Wavelength
  1. Vacuum wavelength of the laser
  2. Refractive index of air determination
    - Refractive index equation
    - Air temperature measurement
    - Atmospheric pressure measurement
    - Humidity measurement
    - Air composition
- Interferometer
  1. Alignment of interferometer axis with scale graduation axis (i.e., Abbe offset)
  2. Structural characteristics
    - Constancy of distance between reference mirror and microscope
    - Constancy of distance between beam splitter and microscope
    - Constancy of distance between measuring retroreflector and scale
- Scale
  1. Temperature measurement
  2. Thermal expansion coefficient
  3. Graduation quality

Except for interferometer structural characteristics, and scale graduation quality, these parameters lead to uncertainties proportional to the scale length being measured.

Relative effects of length-dependent uncertainties are illustrated in Table 3 where it is shown, for example, that a 0.1 μm length measurement uncertainty will result from an uncertainty of 0.009 °C in temperature measurement of a one meter steel scale (assuming a linear thermal expansion coefficient of 11.5 × 10^−6^/°C.

There are two ways of evaluating process uncertainty. First, there is the uncertainty budget method in this table. Uncertainties from each source are in the two right hand columns for the period before (labeled 1986) and after (labeled 1994) the uncertainty re-evaluation. The accepted method for calculating process uncertainty is to add the individual values in quadrature, i.e., take the square root of the sum of their squares. These combined standard uncertainty values (*u*_s_) are at the bottom of the two columns.

The second method is to compare measurements of a control scale performed by different but equally valid measurement methods. This method will be used in Sec. 3.7 where results of international measurements of a meter bar are analyzed to arrive at an uncertainty.

#### 3.6.2 Procedure

The following actions were taken for this study:

- Recalibrated temperature measurement system
- Recalibrated barometer
- Recalibrated hygrometer
- Applied zero-shift (deadpath) corrections to interferometric data.
- Redetermined vacuum wavelength of the laser
- Measured ambient carbon dioxide content of air in the length scale laboratory.
- Re-examined interferometer and scale alignment procedures.
- Evaluated photoelectric microscope setting variability.
- Carried out an international measurement interchange by obtaining, by loan from BIPM, steel meter bar No. 12924, together with its measurement history. Meter bar No. 12924 was used in an international intercomparison organized by BIPM. It was circulated among the major national measurement laboratories from 1976 to the present and it is a valuable tool for evaluating measurement processes because of the quality of its measurement history. It was measured at NBS in l977.
- Tested the recently proposed revision of Edlén’s air refractivity equation.
- Converted from the International Practical Temperature Scale of 1968 (IPTS-68) to the International Temperature Scale of 1990 (ITS-90).

#### 3.6.3 Results

##### 3.6.3.1 Temperature, Pressure, and Humidity

During June through September 1987, the measuring instruments for these parameters were recalibrated. The temperature measurement system was found to have remained within its accepted uncertainty of 0.002 °C. The barometer calibration changed 16 Pa since 1985, equivalent to a fractional length change of 4 × 10^−8^. The hygrometer calibration changed 117 Pa (5 % r.h.) since November 1985, equivalent to a fractional length change of 4 × 10^−8^. Algebraic signs of these changes tended to make their uncertainties cancel, thus reducing the net effect. Undoubtedly the contribution of these uncertainties added to measurement variability and uncertainty by fractional amounts varying from zero to about 4 × 10^−8^ during some of the period from 1985 through most of l987.

No corrections to existing data were made to compensate for these uncertainties, but barometers and hygrometers with better claimed stability were obtained. More frequent checks on these instruments have been made since March 1990. Some instabilities are still being found, but humidity measurement standard uncertainty is now estimated at 28 Pa (1.2 % r.h.), and barometric pressure standard measurement uncertainty is 8 Pa (0.06 mm of Hg). These estimates come from frequent intercomparisons of three hygrometers in the line scale laboratory, two of which have a NIST calibration. For pressure, there are two barometers from different manufacturers, and both of them have a NIST calibration. As soon as divergence among these instruments is detected, new NIST calibrations are obtained.

##### 3.6.3.2 The International Temperature Scale of 1990 (ITS-90)

In 1990 a new International Temperature Scale (ITS-90) [22] was adopted to replace the International Practical Temperature Scale of 1968 (IPTS-68) [23]. The temperature measurement system was recalibrated in September 1991 to reflect the change. The relationship between the two scales at 20 °C is

$$t_{90}\left(20\;{}^{\circ}C\right)\;-t_{68}\left(20\;{}^{\circ}C\right)=\;-0.005\;{}^{\circ}C.$$

During the period between the official adoption of ITS-90 and this recalibration, line-scale measurements were corrected for the difference.

Measurements of steel control bar No. 6495 were made before and after the temperature scale change and recalibration for verification. Since the linear thermal expansion coefficient of this bar is 11.75 × 10^−6^/°C the computed length change from 20 °C IPTS-68 to 20 °C ITS-90 is + 0.030 μm. The measured change was + 0.04 μm with a standard uncertainty of 0.01 μm. The 0.01 mm difference between the measured and computed length change is equivalent to 0.002 °C. This confirms the temperature uncertainty value and the conversion to ITS-90.

##### 3.6.3.3 Carbon Dioxide

**Fig. 13:**
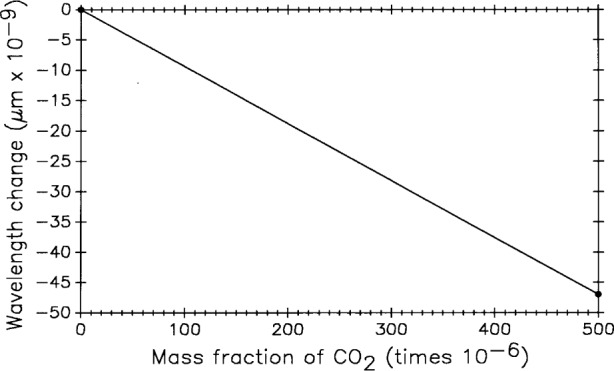
Effect of atmospheric CO_2_ concentration levels, 1958 to 1988.

Carbon dioxide levels were measured in the NIST length scale laboratory in 1990. The mass fraction of ambient CO_2_ with no one in the room averaged 350 × 10^−6^. With one person in the room the average increased to 375 × 10^−6^, and with two people it increased to 400 × 10^−6^. During length scale measurements there is occasionally more than one person in the laboratory so 380 × 10^−6^ (1.2 people) was selected as a reasonable average value. In the Edlén equation [12] for the refractive index of air a value of 300 × 10^−6^ is assumed. Figure 13 shows the relationship between CO_2_ content of air and the 0.6328 mm laser wavelength applying Jones’ [24] analysis of the effect. Raising assumed CO_2_ values from 300 × 10^−6^ to 380 × 10^−6^ changes measurements of a one meter length by − 12 nm.

**Fig. 14:**
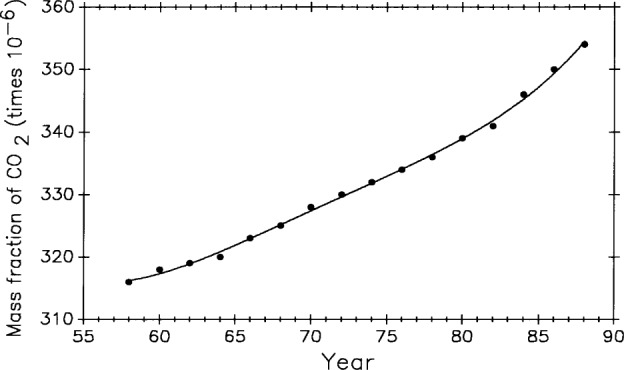
Atmospheric CO_2_ concentration levels, 1958 to 1988.

Figure 14 shows the trend in ambient atmospheric CO_2_ levels according to historical records [25]. From 1958 to 1988 the mass fraction of CO_2_ increased by approximately 40 × 10^−6^. While values may change with the season and from one geographic location to another the chart is representative of a worldwide trend. All values for the control standards have been adjusted by assuming an average laboratory level 30 × 10^−6^ above average ambient and prorating the change over the 26 years of interferometric measurements. Relative standard uncertainty in length meausrement caused by uncertainty in the CO_2_ value is estimated to be 0.01 μm/m.

##### 3.6.3.4 Laser Vacuum Wavelength

A measurement of the laser vacuum wavelength at NIST in 1989 revealed a fractional decrease of 7 × 10^−8^ since 1979. For lack of a better model this change was prorated over the data on M5727 and No. 6495 for the period from 1979 to l989. Laser wavelength measurements are now more readily available than they were in the past so this uncertainty source will be maintained at or below 2 × 10^−8^ by frequent calibrations.

##### 3.6.3.5 Linear Thermal Expansion

Linear thermal expansion coefficients of both control standards are supplied by the manufacturer. A value of 1.23 × 10^−6^/°C is given for Invar meter M5727, and 11.75 × 10^−6^/°C for 0.508 meter steel bar No. 6495. Standard uncertainties in the coefficients do not exceed 0.06 × 10^−6^/°C for M5727, and 0.25 × 10^−6^/°C for No. 6495. Considering that the measuring temperature never deviates more than 0.01 °C from the reference temperature of 20 °C, the potential uncertainty is a negligible 1 nm for both bars.

Uncertainties in temperature measurement and expansion coefficients cannot be taken lightly, however. Under less ideal conditions these uncertainties can result in significant length uncertainties. For example, relative standard uncertainty of 10 % in the coefficient of a steel bar (nominally 11.5 × 10^−6^/°C) at a measuring temperature of 21 °C will result in a length measurement relative standard uncertainty of 1.15 × 10^−6^ when correcting to the standard temperature of 20 °C. Likewise, a temperature measurement uncertainty of 0.1 °C at 20 °C but with no uncertainty in the coefficient will result in the same size uncertainty.

##### 3.6.3.6 Interferometer and Scale Axis Alignment

A more precise method for aligning the scale graduation axis with the interferometer optical axis was devised in mid l987. An existing 3 mm diameter peg on the back of the retroreflector case, located on the retroreflector axis, was lengthened a few millimeters so that it would extend under the microscope objective lens when the carriage was moved all the way to the right. Milling a flat bottomed notch halfway through the extended peg and scribing an axial line at the center of this surface created an axially coincident reference line. With this device in place it takes three steps to complete the alignment: (1) Mount, focus, and align the scale in the microscope field center as the carriage is moved back and forth. The center is defined by a crosshair reticle in the eyepiece. (2) With the extended peg under the microscope, adjust the retroreflector until the reference line is focussed and aligned. It is then coincident with the scale axis. (3) Adjust the laser beam into coincidence as the carriage is moved through its full travel.

Relative standard uncertainty from this critical adjustment is reduced to less than 1 × 10^−8^ by the new procedure.

##### 3.6.3.7 Revised Air Refractivity Equation

The most significant change in Edlén’s equation is in the correction factor for air moisture [14]. Indirect testing of the revised correction was done at NIST by measuring control standard M5727 with the line scale interferometer over a range of partial pressure of water vapor in air from 467 Pa to 1167 Pa (20 % to 55 % r.h. at 20 °C). These measurements were made as part of this study of systematic effects but results were published separately [15].

**Fig. 15:**
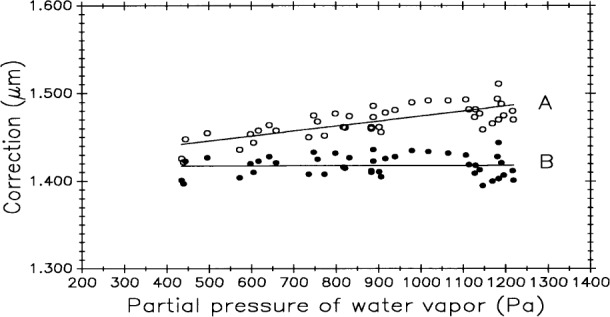
Meter M5727, 0 m to 1 m length vs water vapor content of air.

Test results are graphically summarized in Fig. 15. Measured length values of M5727, computed two different ways, are plotted against partial pressure of water vapor. Plot A is the data computed with the 1966 Edlén equation and plot B is the same data computed with the revised Edlén equation. Plot B shows a reduction in correlation between length and water vapor content and it verifies that the revised equation provides a much better estimate of the refractive index of moist air.

**Fig. 16:**
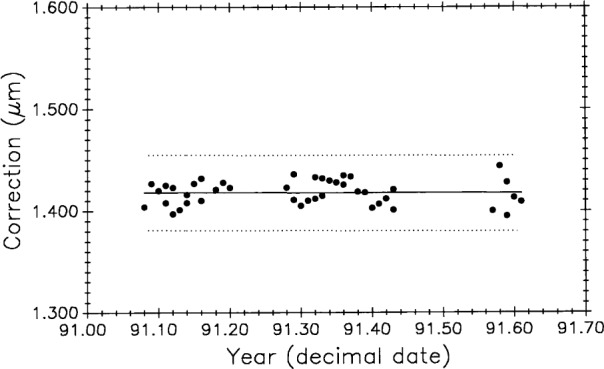
M5727, length vs measurement date in the water vapor experiment.

In addition to confirming the revised water vapor correction, two things are demonstrated and discussed in Refs. [14] and [15]: (1) closer attention must be paid to the accuracy and reliability of the hygrometers, and (2) air moisture content must be measured inside the interferometer housing. Using the revised equation and better air moisture measurement methods improved length measurement variability (3*σ*) from 0.13 μm for the period from 1971 to the end of 1991 to 0.04 μm for the seven month period shown in Fig. 16.

All control data was retroactively adjusted for the change in the water vapor correction, but uncertainties still exist up to period M8 in measuring water vapor because the hygrometer was outside the interferometer housing and, in some cases, the hygrometer was out of calibration. Even so, variability improved from 0.15 μm to the 0.13 μsm value in Fig. 11 by this adjustment.

Relative standard uncertainty in the revised version of the Edlén equation is estimated by the authors [14] to be 3 × 10^−8^ but this includes uncertainties in measuring t, p, and f. The uncertainty 2 ×s 10^−8^ is used in Table 2.

##### 3.6.3.8 Interferometric Zero-Shift Corrections

Beginning in 1989, zero-shift corrections [26] (often called deadpath corrections) were applied to compensate for changes in the refractive index during a measurement. These changes expand or contract the standing wave train between the remote interferometer and the retroreflector and thus affect the interferometric fringe count. Barometric pressure often changes during a measurement and has the greatest influence of the three parameters on the refractive index. Air temperature and moisture content change slowly, if at all, and have a relatively small effect.

The 43 measurements of M5727 in the 1991 Edlén equation experiment [15] provide data for evaluating the zero-shift correction. Table 4 shows mean values and statistics for the one meter interval of M5727 with and without correction. Although the mean length changes by only 2 nm, variability is reduced by more than a factor of two. Algebraic signs of the corrections change with direction of pressure change and with direction of interferometric counting, so over the long term, zero-shift corrections tend to cancel and have minimal effect on mean length. However, making the corrections improves both short and long term variability.

##### 3.6.3.9 Correlation Test: Length vs Barometric Pressure

**Fig. 17:**
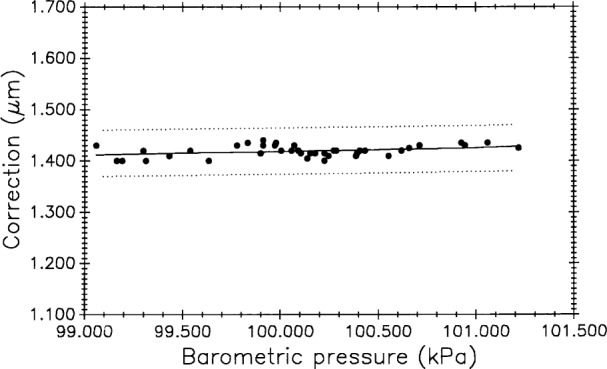
M5727, length vs barometric pressure correlation test.

If a correlation exists between control standard length and atmospheric pressure it could indicate further problems with the wavelength correction equation. Figure 17 shows data from the air moisture experiment (plot B, Fig. 15) with corrections to the one meter length of M5727 plotted against observed barometric pressure. No statistically significant correlation is indicated.

##### 3.6.3.10 Summary of Data Adjustments

All measurement data on both controls were adjusted retroactively for the following changes:

1. The international temperature scale change from IPTS-68 to ITS-90.
2. Laser vacuum wavelength change between 1979 and 1989.
3. Carbon dioxide content change of laboratory air from 1966 to the present.
4. Adoption of the revised refractive index of air equation.

Control data were adjusted for interferometric zero-shift starting in 1989, a procedure that is now standard practice.

### 3.7 International Measurements: BIPM Meter Bar No. 12924

The second method for evaluating uncertainty from systematic effects mentioned in Sec. 3.6.2 is to compare measurements of a scale as performed by several independent and equally valid methods. This was done through the good offices and efforts of the Bureau International des Poids et Measures (BIPM). Starting in 1976, BIPM sent steel meter bar, SIP No. 12924, to the national standards laboratories of most of the industrialized nations in a successful and useful international standardization effort.

The following laboratories measured this bar: (Note: Country and laboratory names are those at the time measurements were made.)

1. Bureau International des Poids et Mesures (BIPM), Sèvres, France
2. National Measurement Laboratory (NML), Lindfield, Australia
3. National Research Council (NRC), Ottawa, Canada
4. National Bureau of Standards (NBS), later NIST, Washington, DC, USA
5. National Research Laboratory of Metrology (NRLM), Ibaraki, Japan
6. National Physical Laboratory of (NPL), Teddington, UK
7. Amt für Standardisierung, Messwesen und Waren-prufüng (ASMW), Berlin, East Germany
8. Institut Métrologie D. I. Mendéléev (INM), Leningrad, USSR
9. Physikalisch-Technische Bundesanstalt (PTB), Braunschweig, West Germany
10. Instituto di Metrologia G. Colonnetti (IMGC), Turino, Italy
11. National Institute of Metrology (NIM), Beijing, PRC
12. Federal Office of Metrology (OFMET), Wa¨bern, Switzerland

**Fig. 18:**
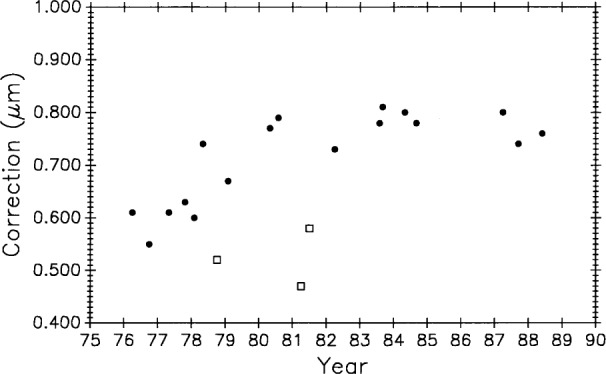
International measurement data on BIPM Meter No. 12924, 0 m to 1 m.

BIPM measured the bar six times, NBS/NIST three times, NML and IMM twice each, and the remaining laboratories once each for a total of 21 measurements over a period of 12 years. Values for the one meter length are shown graphically in Fig. 18.

**Fig. 19:**
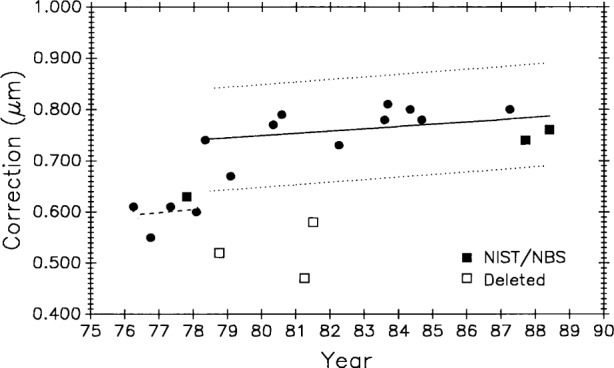
Lines fitted to the data on No. 12924, 0 m to 1 m.

In evaluating these data [27], BIPM concluded from its own and other measurements that the bar experienced a sudden lengthening of over 0.1 μm early in 1978. Such a change was probably caused by a severe mechanical shock during shipment. Based on this conclusion the data were divided into two groups; one before and one after the change. BIPM further concluded that the bar has a long term linear growth trend. This is shown in Fig. 19 where linear fits are made to the two groups of points. The first group lacks sufficient data and time span to establish a slope so it was given the same slope as the second group. Three points, indicated on the graph by diamonds, were deleted from the analysis. When they were included in the data the ±s 3*σ* limit becomes much larger and they could not be rejected by the accepted statistical test. In this case they were rejected judgementally because they did not fit the pattern convincingly established by the other 12 points in the second group.

NBS/NIST measurements are indicated in Fig. 19 by squares. The 1987 and 1988 points agree with the international average, as represented by the line fitted to all the accepted points, within 0.04 μm and 0.03 μm (0.04 μm/m and 0.03 μm/m), respectively. Control bar M5727 was measured a number of times during this same period so the validity of these M5727 measurements is greatly enhanced, the stability of the NIST measurement process is verified, and the case for growth in M5727 is strengthened. Table 5 shows these values.

### 3.8 Interpreting Control Charts

#### 3.8.1 Secular Change, 1971 to 1991

Long-term length changes have occurred in both control standards. Using all 68 data points for M5727 (Fig. 11) from 1971 to 1991 in a linear regression shows a positive growth rate (slope) of 0.0115 (μm/m)/a.

The 0.508 m control No. 6495 (Fig. 12) shows negative growth rate of 0.0196 (μm/m)/a.

It is interesting that one control is growing and the other is shrinking with time. The processes that cause secular dimensional changes in these two bars are obviously different.

#### 3.8.2 Control Charts Incorporating 1991-98 Data

**Fig. 20:**
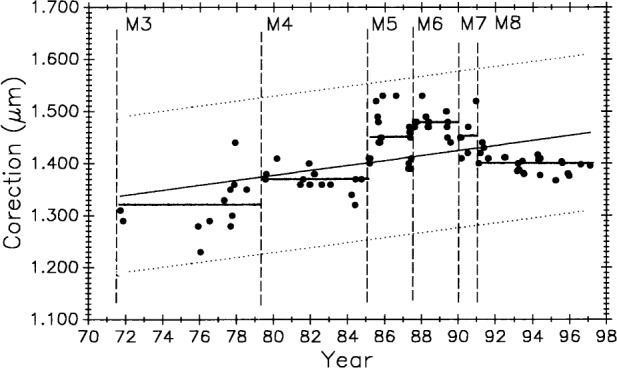
Meter M5727 control chart, 0 m to 1 m, 1971 to 1998.

**Fig. 21:**
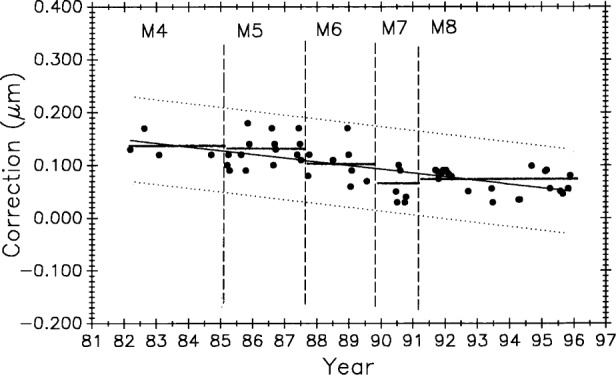
Control chart for bar No. 6495, 0 m to 0.508 m, 1982 to 1996.

Figures 20 and 21 are the control charts for M5727 and No. 6495 with recent (M8) data included. The mean value for each period is shown as a horizontal line in addition to the line fitted to all the plotted data.

Of the 134 data points available on M5727, 43 (32 %) are in M8. Plotting all 43 points in M8 will give excessive weight to this group. Since 1976 an average of five data points per year were taken. On that basis, five points from the M8 period is a reasonable weighting of the data. Five points were selected to give the same mean value and approximately the same spread as the 43 points. The slope of the linear fit to all the plotted M5727 data in Fig. 20 shows a growth of 0.0052 (μm/m)/a.

The 8 points in M8 for No. 6495 are not weighted. The slope of the fit to all the No. 6495 data in Fig. 21 is a negative 0.0120 (μm/m)/a.

The effect of M8 data on slope is pronounced, especially on M5727. This evidence strongly suggests that data taken since 1991 should be treated as representing a new measurement process. More will be said about his in Sec. 3.9.

In Table 6, measurement variability for both controls is shown for each period.

Changes in variability following M4 (i.e., M5, M6, M7) were probably caused less by planned process changes than by factors such as undetected uncertainties in the barometer and hygrometer, variations in interferometer alignment, and uncertainties in air moisture measurements caused by the hygrometer being outside the interferometer housing. Period M8, however, shows a major shift in the measurement process brought about by adopting the revised Edlén equation and improved air moisture measurements. These length dependent effects on M5727 are double what they are on No. 6495.

#### 3.8.3 Simulating Stability in Control Standards

**Fig. 22:**
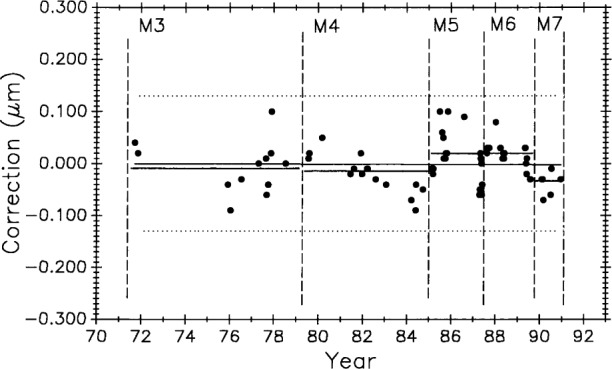
Simulated stability for M5727, 0 m to 1 m, with mean value for each period.

Figure 22 is the 1971 to 1991 data on M5727 corrected for secular length change. It shows the mean for each period (individual horizontal solid lines) in addition to the continuous horizontal line fitted to all the data. This chart is produced by plotting the residuals, i.e., observed minus computed values, based on Eq. (9) for the fitted line in Fig. 11. If the control standard had remained constant in length through this time period its measurement history would look like Fig. 22. Simulated stability makes evaluating process changes easier by releasing group mean values from data slope influence (compare with Fig. 11). Viewed in this way none of the process changes make a significant change in mean length. This demonstrates that the measurement process is consistent throughout its 25 year history despite changes made in the process.

**Fig. 23:**
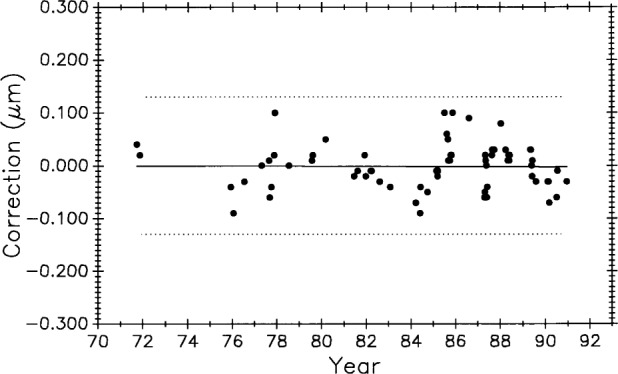
Simulated stability for M5727 with data treated as one group.

Figure 23 is similar to Fig. 22 except that the data are seen as a single group. The basic assumption in most statistical techniques is that the data are a random sample from a stable probability distribution and in most cases a normal frequency distribution is formed. Stability in this case is simulated by the secular change adjustment.

**Fig. 24:**
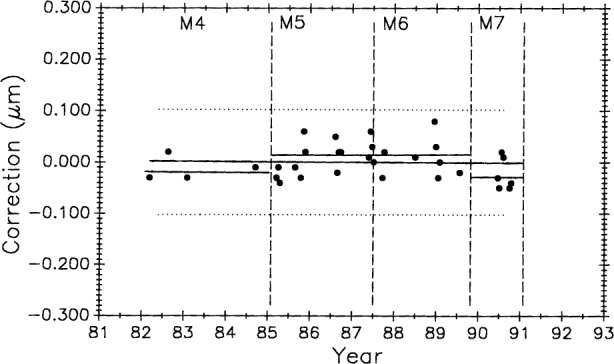
Simulated stability for bar No. 6495 with mean value for each period.

**Fig. 25:**
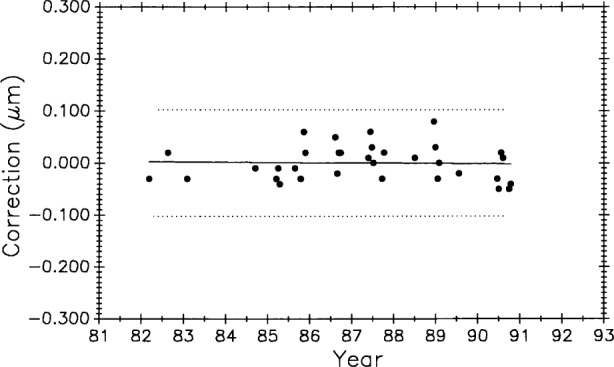
Simulated stability for bar No. 6495 with data treated as one group.

In the same manner Fig. 24 shows simulated stability data on control bar No. 6495 with a mean value for each period. In Fig. 25 the data is treated as a single group.

**Fig. 26:**
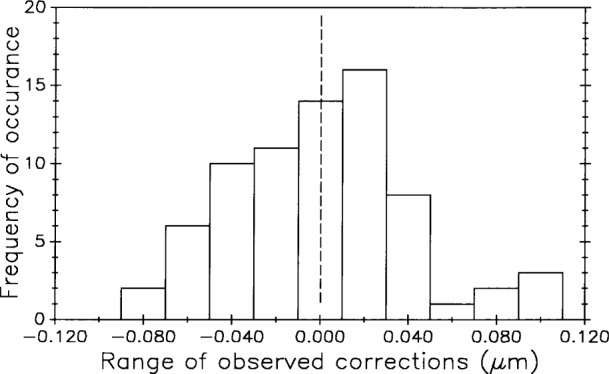
Histogram of simulated stability data on M5727.

The histogram in Fig. 26 tests how closely the data approximates a normal distribution. It peaks near the zero value as it should, but its lack of perfect symmetry probably indicates insufficient data (there are 120 points) rather than bias.

#### 3.8.4 The Measurement Process in an Out-of-Control State

Three times the standard deviation of a single observed value predicted by a fitted line is an accepted criterion for judging the performance of a process. There is a 99.7 % chance that the next measurement value will fall within the ± 3 *σ* control limits if the process is in statistical control. When a value falls outside these control limits the process is said to be out of control. If the cause of the out-of-control measurement is found, or if the next measurements are back in control, the errant value can be deleted. If out-of-control measurements persist, the cause must be found and corrected before any values can be deleted. During periods when the process is out of control, uncertainty of measurements made with the process must be increased or not stated at all. Statistical control limits are periodically recomputed, as data accumulates, to reflect changes in process variability. Frequent measurement of control standards will detect problems when they arise and ensure that the process is performing as predicted.

For a time in 1986, the process did go out of control. A series of erratic values on M5727, many of which fell outside the existing control limits, had a ± 3*σ* value exceeding 0.4 μm. It took more than a week to find that the retroreflector mount on the carriage had loosened and was shifting slightly during measurements. Once the mount was tightened, control was re-established and out-of-control data deleted.

#### 3.8.5 Control Standard Subintervals

**Fig. 27:**
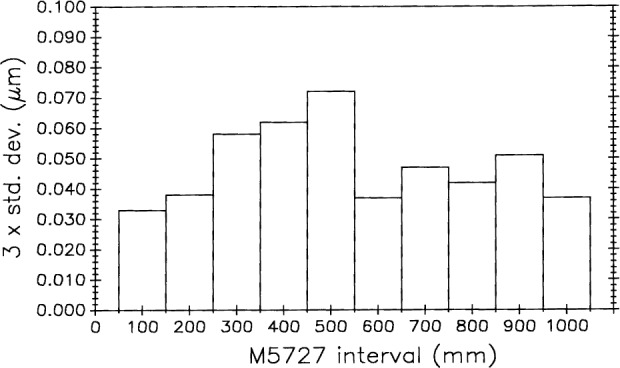
Measurement variability for M5727 decimeter subintervals.

Figure 27 shows the variation in measurement variability among the M5727 decimeter subintervals in the water vapor compensation experiment (plot B in Fig. 15).

Lack of sharp focus influences variability. During set-up procedures scales are supported at two points (Bessell points in this case to produce minimum bending) and to ensure a reproducible condition. The terminal graduations at each end of the scale are brought into sharp focus, but there may be degradation of focus on subinterval graduations if the scale surface is not flat. Depth of focus in the objective lens is also a factor. Because of manufacturing difficulties, long scales are more likely than short ones to be less than perfectly flat.

Other effects in addition to lack of sharp focus can influence subinterval variability. For example, there may be slight variations in graduation symmetry or there may be blemishes on or near the graduations that can influence photoelectric microscope centering. On M5727 the 500 mm graduation has both a blemish near it and a slight loss of focus.

### 3.9 Treating Period M8 Data as a New Process

**Fig. 28:**
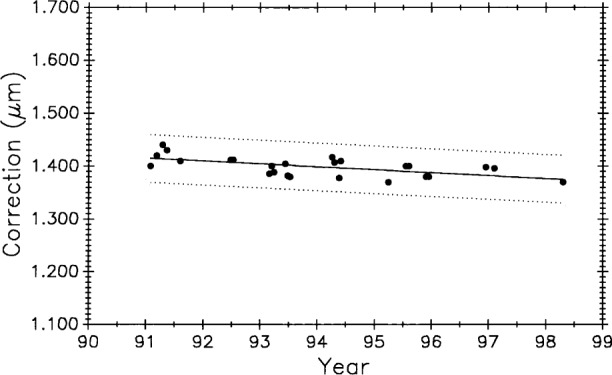
M5727 data, 1991 to 1999, establishes new measurement process.

**Fig. 29:**
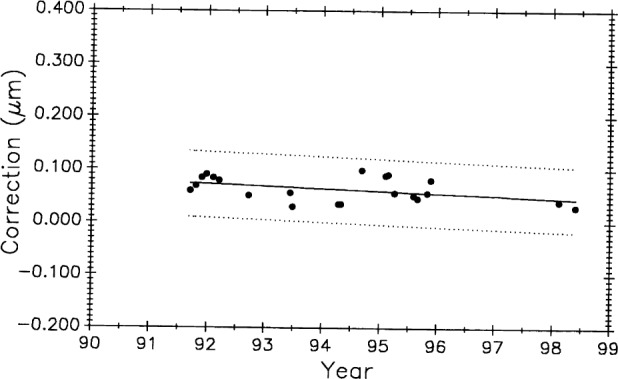
No. 6495 data from new process, 1991 to 1999.

Data taken after coupling and kinematic mounting of the microscope and interferometer components in 1971 was treated as a new process (see Sec. 3.5.1). Now there is compelling evidence justifying that data taken since 1991, when the revised Edlén equation was adopted, should be treated as a new measurement process. Measurement values and variability have changed on both controls, as shown in Figs. 28 and 29, but it is less apparent in No. 6495 because it is influenced less by length dependent uncertainties except temperature. The rate of change derived from a linear fit to the data on M5727 is − 0.0059 (μm/m)/a. For No. 6495 the rate is − 0.0086 (μm/m)/a.

The slope of the fit is fairly uniform for the various data sets on the 0.508 m bar. However, it is not uniform for M5727 which now shows a negative slope for the first time. Secular dimensional behavior of materials varies widely so this is probably attributable to the metallurgy of the Invar alloy.

For the last seven years the new process has performed consistently and with less variability than before. Fig. 23 shows the old process with the ± 3*σ* control limits at ± 0.13 mm while the new process is shown in Fig. 28 with the control limits at ± 0.044 mm. See Table 7 for the long term history of variability on both controls.

### 3.10 Measurement of Short Intervals

**Fig. 30:**
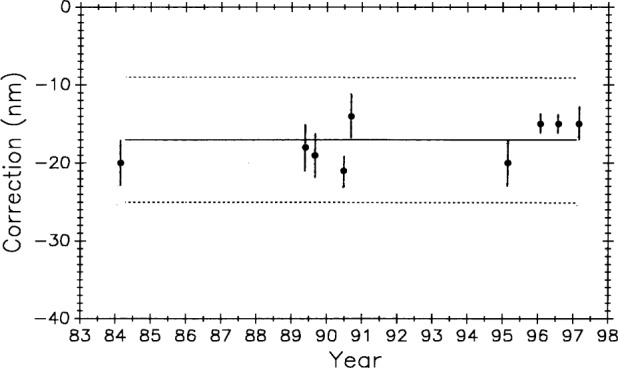
SRM474, 0 mm to 4 mm control, 1984 to 1998.

Demand for short interval measurements has risen with the development of ever smaller electronic circuitry. Scales ranging from a few micrometers to a few millimeters with line spacings from 1 μm to 10 μm are in this category. Measurement results on a 4 mm interval on the short-interval control standard, SRM474, are shown in Fig. 30 for the period from 1984 into 1997.

Each point is the mean of at least 10 measurements. Expanded uncertainty for each point is shown by a vertical line through the point. All measurements are corrected for zero-shift. Based on these data, the ± 3*σ* control limits are ± 8 nm, but starting in 1996 the data shows marked improvement in variability. This shortinterval process, like that for longer lengths, has improved and warrants being treated as a new process. Length dependent uncertainties drop out except for the zero-shift correction, and zero-shift can be minimized by keeping the deadpath as short as possible and making measurements during periods of steady atmospheric pressure. Expanded uncertainties of one nanometer are now justified when the statistical analysis confirms them. This has been verified by several short interval calibrations for customers where one nanometer expanded uncertainty was achieved.

### 3.11 Long Term Variability

Table 7 shows long term variability as represented by control chart limits taken from Figs. 23, 28, and 29. Secular change is eliminated as a factor and the water vapor correction uncertainty is partially reduced in Fig. 23, and greatly reduced in Figs. 28 and 29. The most likely causes of the limits prior to 1991 are variations in instrument calibrations (particularly the barometer and hygrometer), variations in alignment of the interferometer, unstable barometric pressure caused by weather conditions, gradual or sudden changes in laser vacuum wavelength, or a temporary mechanical distortion in the apparatus. These problems are mitigated in measurements since 1991 by the attention given to factors causing them.

### 3.12 Measurement Uncertainty

Until recently there was no universally accepted method for evaluating and stating measurement uncertainty. Various international organizations, led by the Comité International des Poids et Mesures (CIPM), have addressed this problem. The outcome is an International Standards Organization (ISO) document, *Guide to the Expression of Uncertainty in Measurement* [28]. NIST policy based on the ISO document is set forth in NIST Technical Note 1297, *Guidelines for Evaluating and Expressing the Uncertainty of NIST Measurement Results* [21].

This protocol divides uncertainties into two categories:

- Those uncertainties evaluated by statistical methods;
- Those uncertainties which are evaluated by other means. In the line scale measurement process, for lengths greater than 10 mm, Type B uncertainties tend to predominate and each one is evaluated independently. They are combined and summarized in Table 3, and verified by the BIPM meter bar measurements (see Sec. 3.7). The value of 5 × 10^−8^ is length dependent and is called standard uncertainty arising from systematic effects, *u*_s_ (Type B).

When a high quality scale is calibrated it is measured with at least eight passes from which a standard deviation is computed for each interval. Variability represented by this standard deviation is Type A uncertainty and it comes from mostly nonlength dependent uncertainty sources. These sources are in the microscope and its line centering servo system, the interferometer system, humidity and barometric pressure instruments, graduation quality, and in critical distance changes between retroreflector and scale, and between microscope and remote interferometer. These components cannot be evaluated separately during a scale calibration procedure. Their combined value, contained in the standard deviation, is called standard uncertainty arising from random effects, *u*_r_ (Type A).

For line scales a typical Report of Calibration uncertainty statement is: “Each length value is the mean of eight measurements and the expanded uncertainty in each value is

$$U=2\sqrt{{\left(u_{r}\right)}^{2}+{\left(L\times u_{s}\right)}^{2}}$$

where *L* is the nominal interval length in meters and 2 is the coverage factor.”

### 3.13 Conclusions

This effort to reduce uncertainties has improved measurement control chart limits (variability), both short and long term, and it lowered measurement uncertainty. Improvements were significant or noteworthy in the following cases:

- The revised air refractivity equation produced the most dramatic improvements in variability and uncertainty.
- Improved hygrometric measurements contributed substantially to the improvements.
- Barometric pressure measurements are now more reliable but an expanded uncertainty of 8 Pa (0.06 mm of Hg) remains. We believe this can be improved with better instrumentation and calibration.
- Correcting for interferometric zero-shift (deadpath correction) has little effect on long term values but improves short term and long term variability.
- The improved interferometer and scale axis alignment method, although not yet proven, should reduce these uncertainties.
- Because laser vacuum wavelength measurements are now more readily available, uncertainties from this source are reduced.

Finally, interchanges of the BIPM meter bar verify that the NIST line scale measurement process is consistent and well within the control limits of the world’s premier national laboratories. Simulation of control bar stability further demonstrates process stability. Process relative standard uncertainty is now estimated to be 5 × 10^−8^ by the uncertainty budget method (see Table 3) and 3.5 × 10^−8^ by the independent measurements method (international round robin). This is a marked improvement over the 9 × 10^−8^ value in 1986.

## 4. Future Plans

### 4.1 Increase Line Detection Resolution

Although nanometer resolution is achievable with the present optical system under ideal conditions, there is the potential for further improvement with other detectors. One of these is the line scanning capacitance detector now under development. It offers the most promising approach to improving line detecting resolution.

### 4.2 Upgrade the Displacement Interferometers

A new high resolution interferometer system is being installed at the opposite end of the waybed from the present system. When complete, there will be two interferometer systems in place on a permanent basis. This was done on a temporary basis at various times in the past. Two systems can be used simultaneously, separately, or differentially for various applications. An opportunity exists for offering an intercomparison service for interferometer systems with the dual operation feature.

### 4.3 Upgrade the Data Acquisition and Control Computer Systems and Their Interfaces

Along with a new interferometer system comes the installation of a state-of-the-art computer/controller. This will provide faster, more flexible, and more auto mated operation. For example, temperature, barometric pressure, and humidity data will be acquired at every measurement interval on a scale. This data will be used to compute and correct each interval for the exact conditions at the moment of measurement and provide automatic zero-shift corrections.

### 4.4 Improve the Mechanical Structure

Many structural changes were made over the years, and more are in the planning stage. Foremost among these is a plan to use low thermal expansion Invar in the beams coupling the interferometers to the line detector. These beams, one in each direction from the line detector, and each about 1 m long are steel at present. Using low thermal expansion material will reduce changes in critical dimensions between the line detector and the two remote interferometers located at the ends of the beams. Temperature changes occur slowly in the beams but can be significant in lengthy scale calibrations.

## 5. Summary

The NIST Length Scale Interferometer has served well, and is still serving well, as both a research tool and a calibration instrument. By systematic performance monitoring with a measurement assurance program, and incremental improvements through experimentation, this instrument keeps abreast of accelerating national accuracy requirements. It proved itself in international intercomparisons to be on a par with the best national laboratories. There will continue to be a need for high accuracy line spacing measurements and it is likely that the line scale interferometer with its proposed improvements will fill that need.

## Figures and Tables

**Fig. 2a:**
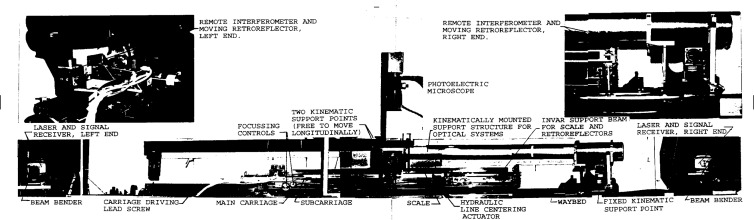
Photograph of the length scale interferometer in its present form.

**Fig. 2b:**
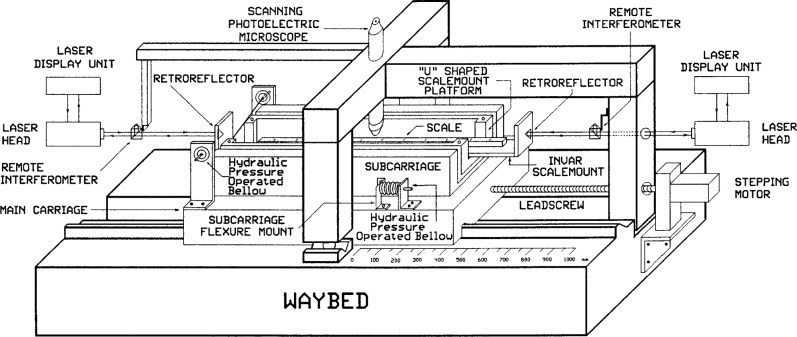
Schematic diagram of the length scale interferometer in its present form.

**Fig. 9a:**
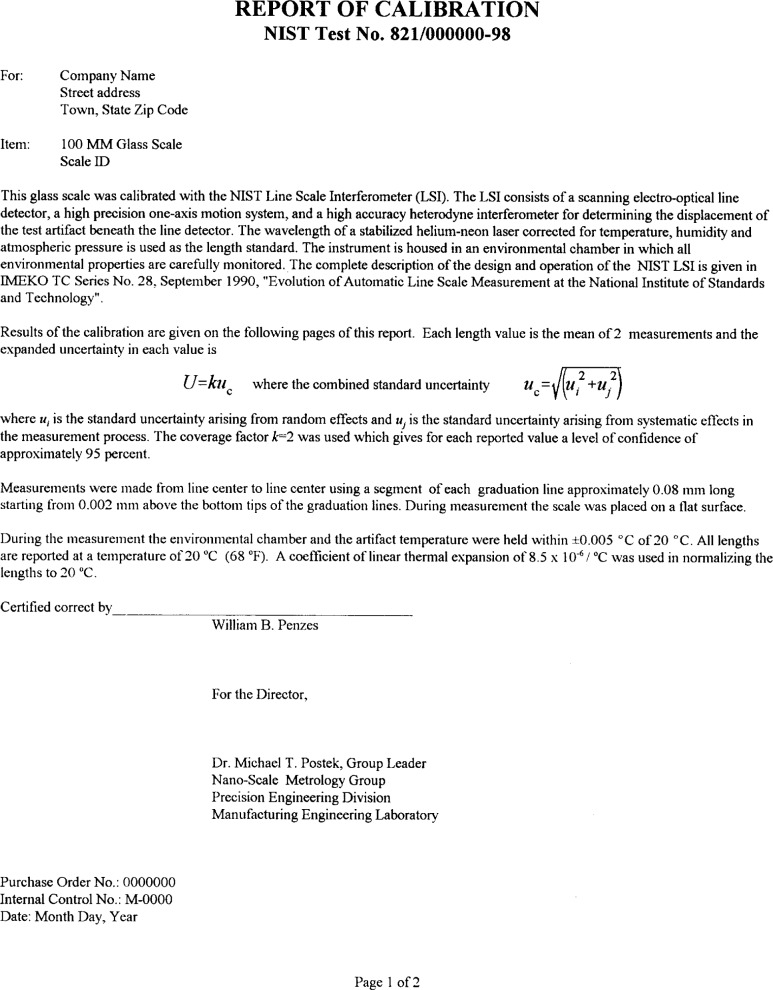
Typical line scale calibration report (page 1).

**Fig. 9b:**
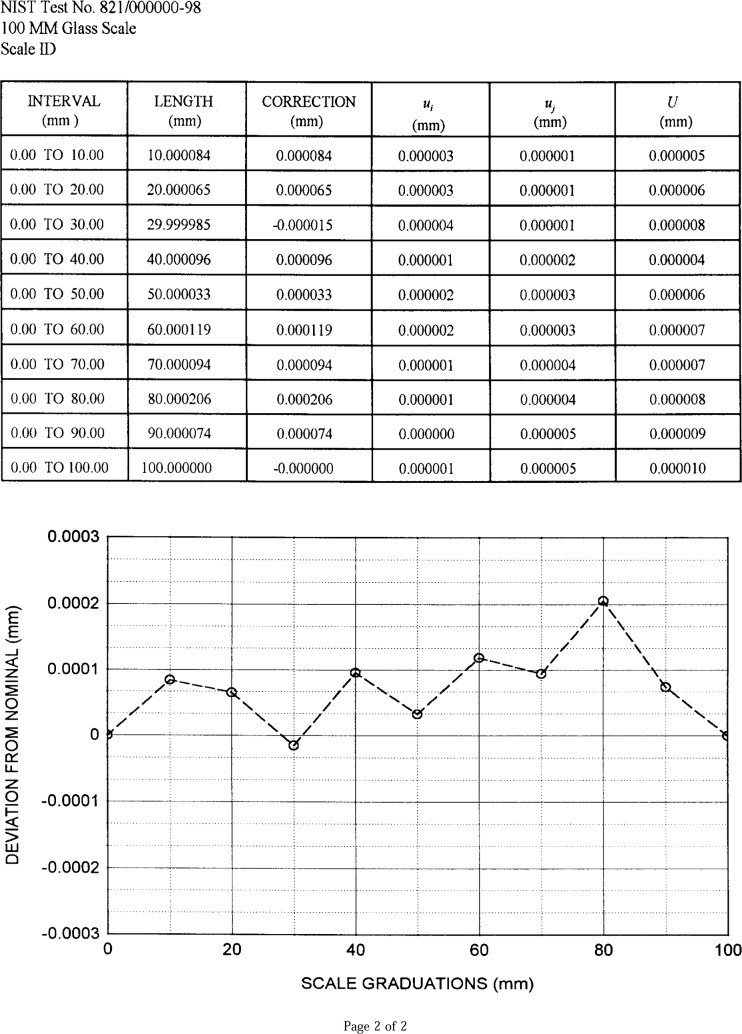
Typical line scale calibration report (page 2).

**Table 1:** Sequence and descriptions of process changes

Period symbol	Period descriptions
M1	Mean of data from classical meter bar intercomparisons (individual points not shown).
M2	Data from the first interferometric measurements.
M3	Data taken after the microscope, beam splitter and referencemirror were rigidly coupled forming an assembly that was kinematically mounted on the waybed. This change was designed to reduce the effect of the heavy moving carriage on the critical dimension between microscope and beamsplitter.
M4	Data taken after replacing the original Michelson type interferometer with a commercial laser interferometer system mounted at the opposite end of the waybed. No carriage pitch or yaw corrections were made after this change, but scales are now mounted with their ruled axes coincident with the interferome-ter axis to eliminate Abbé offset and its attendant uncertainties.
M5	Data taken after the interferometer retroreflector was moved from its mount on the subcarriage to a mount on the scale support structure (see Fig. 2). This reduced the possibility of change, during a scale measurement, in the critical distance between scale and retroreflector.
M6	Data taken after recalibrating the barometer and temperature system, and replacing the hygrometer.
M7	Data taken after the retroreflector was mounted on a new Invar scale support structure to further reduce chances of a critical distance change. Zero-shift (deadpath) corrections were applied during this and subsequent periods.
M8	Data taken after incorporating a modified version of Edlén’s refractive index equation into the process and mounting a hygrometer inside the interferometer housing.

**Table 2:** Mean value and control chart limits for periods M1 through M4

Period	Mean value (μm)	Control chart limits ± 3*σ* (μm)
M1	1.33	0.24
M2	1.10	0.23
M3	1.32	0.16
M4	1.37	0.07

**Table 3:** Relationship between length-dependent standard uncertainties and standard uncertainties in measurement parameters and estimated process standard uncertainty

Parameter	Relative standard uncertainty in a 1 m length		Process relative standard uncert. 0.01 μ/m	
	for 0.1 μm/m	for 0.01 μm/m	1986	1994
*Wavelength*				
Vacuum wavelength	1 × 10^−7^	1 × 10^−8^	5	2
Refract. index eq.	1 × 10^−7^	1 × 10^−8^	5	2
Air temp.	0.1 °C	0.01 °C	0	0
Pressure	40 Pa	4 Pa	4	2
Rel. humidity	12 % rh	1.2 % rh	3	1
CO_2_ content		67 × 10^−6^	2	1
*Interferometer*				
Alignment	0.45 mm/m	0.14 mm/m	2	2
*Scale*				
Steel temp.	0.009 °C	0.001 °C	2	2
Glass temp.	0.012 °C	0.001 °C		
Invar temp.	0.067 °C	0.007 °C		
Quartz temp.	0.250 °C	0.025 °C		
				
			*u*_s_ = 9	5

**Table 4:** Effect of making zero-shift corrections

Mode	Deviation from nominal length (μm)	Control limits ± 3*σ* (μm)
Without correction	1.420	0.080
With correction	1.418	0.038

**Table 5:** Measurements of control bar M5727 made during same period as measurements of meter No. 12924

Decimalized date (in years from 1900)	M5727 Correction to 1 m interval (in μm at 20 °C)
77.77	1.35
87.67	1.48
88.35	1.48

**Table 6:** Control chart limits (± 3*σ*) for each time period

Period symbol	M5727 0 m to 1 m (μm)	No. 6495 0 m to 0.508 m (μm)
M1	0.24	
M2	0.23	
M3	0.16	
M4	0.07	0.07
M5	0.14	0.08
M6	0.06	0.10
M7	0.10	0.09
M8	0.044	0.062

**Table 7:** Long term control chart limits (± 3 *σ*)

Time period	M5727 (μm)	No. 6945 (μm)
1971–91	0.13	NA
1982–91	0.13	0.08
1991–99	0.044	0.062

## Footnotes

1 A static interferometer is defined as one where none of the optical components are intended to move during a measurement and the interference pattern is stationary. A dynamic interferometer is one where an optical component, usually a retroreflector, moves and the displacement of the moving element is measured by interference fringe counting or its equivalent.

2 Certain commercial equipment, instruments, or materials are identified in this paper to foster understanding. Such identification does not imply recommendation or endorsement by the National Institute of Standards and Technology, nor does it imply that the materials or equipment identified are necessarily the best available for the purpose.

3 These conventions will be followed throughout for control charts.
