# Erratum: The NIST Length Scale Interferometer

**DOI:** 10.6028/jres.105.060

**Published:** 2000-10-01

**Authors:** John S. Beers, William B. Penzes

**Affiliations:** National Institute of Standards and Technology, Gaithersburg, MD 20899-0001

[J. Res. Natl. Inst. Stand. Technol. Volume 104, Number 3, May–June 1999, p. 225]

The caption for Fig. 13 on p. 243 should be **Fig. 13.** Effect of atmospheric CO_2_ concentration on laser wavelength.

